# Computer-Based Connected-Text Training of Speech-in-Noise Perception for Cochlear Implant Users

**DOI:** 10.1177/2331216519843878

**Published:** 2019-04-23

**Authors:** Tim Green, Andrew Faulkner, Stuart Rosen

**Affiliations:** 1 Speech, Hearing and Phonetic Sciences, University College London, London, UK

**Keywords:** cochlear implants, speech perception, noise, training

## Abstract

An interactive method for training speech perception in noise was assessed with adult cochlear implant users. The method employed recordings of connected narratives divided into phrases of 4 to 10 words, presented in babble. After each phrase, the listener identified key words from the phrase from among similar sounding foil words. Nine postlingually deafened adult cochlear implant users carried out 12 hr of training over a 4-week period. Training was carried out at home on tablet computers. The primary outcome measure was sentence recognition in babble. Vowel and consonant identification in speech-shaped noise were also assessed, along with digit span in noise, intended as a measure of some important underlying cognitive abilities. Talkers for speech tests were different from those used in training. To control for procedural learning, the test battery was administered repeatedly prior to training. Performance was assessed immediately after training and again after a further 4 weeks during which no training occurred. Sentence recognition in babble improved significantly after training, with an improvement in speech reception threshold of approximately 2 dB, which was maintained at the 4-week follow-up. There was little evidence of improvement in the other measures. It appears that the method has potential as a clinical intervention. However, the underlying sources of improvement and the extent to which benefits generalize to real-world situations remain to be determined.

## Introduction

The patterns of neural activation elicited by electrical stimulation in cochlear implant (CI) users differ markedly from those in normal hearing. Important limitations include much reduced spectral resolution, changes in temporal firing patterns, and distortion of the mapping between acoustic frequency and cochlear location. While many CI users obtain good speech perception in quiet, such limitations mean that speech in noise is typically much more problematic. For postlingually deafened CI users, there is the additional complication of learning to map degraded and distorted speech information onto stored speech representations established via normal or relatively normal hearing. Reflecting the difficulties involved in such adjustments, speech recognition performance has been found to continue to improve over periods of several months, and even years, after implantation (Ruffin et al., 2007; Tyler, Parkinson, Woodworth, Lowder, & Gantz, 1997). These improvements occur in the context of everyday experience of listening via a CI and may reflect the involvement of cognitive skills, such as maximizing the use of contextual information, in addition to adaptation of purely perceptual processes. One important question is the extent to which formal training might facilitate the adjustment to CI input, particularly since recent advances in technology make it possible for training to be undertaken without the high demands on time and resources involved in face-to-face training.

Recent studies have provided encouraging evidence of the potential efficacy of moderate amounts of various forms of computer-based speech training, for both speech in quiet (Fu, Galvin, Wang, & Nogaki, 2005; Miller, Zhang, & Nelson, 2016; Stacey et al., 2010; Wu, Yang, Lin, & Fu, 2007) and speech in noise (Ingvalson, Lee, Fiebig, & Wong, 2013; Oba, Fu, & Galvin, 2011; Schumann, Serman, Gefeller, & Hoppe, 2015; Zhang, Dorman, Fu, & Spahr, 2012). Important positive aspects of the outcomes of these studies include generalization of improvement across speech materials and talkers, and evidence of benefits being maintained over some considerable time after active training ceased. However, benefits of training have been inconsistent across different studies and across different outcome measures within studies. Of particular concern is that there has been less consistent evidence of improvement for sentence recognition tasks, which more closely reflect real-world communication than tasks requiring phoneme or digit identification.

Several factors may contribute to variability across training study outcomes, including participant factors such as time of onset of deafness and length of time postimplantation, and details of the training task and assessment methods. One potentially important task-related factor concerns the distinction between bottom-up (or “analytic”) and top-down (or “synthetic”) approaches to training. Most previous CI training studies have used a largely bottom-up approach, focusing on the discrimination of particular acoustic-phonetic features, with the underlying assumption that training can alter the neural representations of such features and thereby lead to improved speech perception. Top-down approaches, in contrast, emphasize the development of cognitive and attentional skills and the use of contextual information (Tremblay & Moore, 2012).

Faulkner, Green, and Rosen (2012) have shown that for normal hearing listeners presented with spectrally shifted noise-vocoded speech, a form of computer-controlled interactive training using recordings of connected texts was as effective in improving sentence perception as the highly effective, but labor-intensive, live-voice Connected Discourse Tracking method (De Filippo & Scott, 1978). A connected text, such as a short story, was divided into consecutively presented phrases. After hearing each phrase, listeners were required to select target words from the phrase from among similar sounding alternatives. This procedure, which extended an approach implemented by Stacey and Summerfield (2008), has both bottom-up aspects targeting the learning of phonetic contrasts and top-down aspects involving the use of contextual and semantic knowledge. In addition, the use of a connected text with an on-going narrative may help maintain interest in the task. As Levitt, Oden, Simon, Noack, and Lotze (2011) observed, keeping the trainee entertained can play an important role in promoting commitment to training. Here, we assess whether this computer-based connected-text training can improve speech in noise perception in postlingually deafened adult CI users.

## Methods

### Participants

Nine postlingually deafened, adult, unilateral CI users took part. Table 1 contains details of each participant’s age, CI type, duration of CI use, and pretraining sentence recognition in quiet. To ensure that performance in noise could be measured reliably, participants were required to have scores in quiet of at least 80% on BKB sentences (Bench, Kowal, & Bamford, 1979). Three participants (S2, S7, and S9) used a hearing aid on their unimplanted ear. Ethical approval was granted by the National Health Service Health Research Authority (Ref: 14/LO/0586) and all participants provided informed written consent. Two additional participants withdrew from the study during the pretraining phase, one due to illness and one due to a change in personal circumstances.

**Table 1.** Participant Information.

ID	Age	Implant type	CI experience (months)	IEEE score in quiet (%)	BKB score in quiet (%)
S1	69	Nucleus CP920	32	85	99
S2	53	AB Q90 Naida	16	88	98
S3	70	Nucleus CP910	12	86	99
S4	59	AB Q90 Naida	10	79	90
S5	48	AB Q90 Naida	10	63	97
S6	69	Nucleus 5	10	82	86
S7	65	AB Q70 Naida	97	95	93
S8	60	Nucleus 5	24	80	95
S9	52	AB Q70 Naida	48	99	99

*Note.* AB = Advanced Bionics.

### Training Method

#### Materials

One female and one male talker of standard Southern British English were recorded reading each of three training texts. The texts were graded readers for students of English and therefore had consistent complexity and controlled vocabulary and syntax (Bloese, 2005; Hardcastle, 1975; Revell, 2008). Each text was divided into phrases of 2 to 10 words. The number of phrases per text ranged from 1,034 to 2,641. The median phrase length for each text was five words. For each phrase, between one and four (median = 3) potential target words were selected. Target words were primarily content words, although function words were used in a small proportion of phrases. Similar sounding foil words were chosen for each target. Foils typically shared at least two phonemes with the target and, as far as possible, were chosen so as to be plausible in the context of the narrative. For phrases in which there was only a single potential target word (approximately 5% of the total), a single foil word was chosen. In all other cases, each potential target word had two foils. As an example, for the phrase “The shop was almost empty,” the potential target words were “shop,” “almost,” and “empty” and the foils were “ship,” “stop,” “‘although,” “always,” “entry,” and “twenty.”

#### Task

The participant listened to consecutive phrases presented in 20-talker babble and, following the offset of each phrase, was presented with a display containing target words along with a number of foils. In Faulkner et al. (2012), all of the target words from a phrase were displayed, along with a foil for each target word. This approach was modified in an attempt to reduce the possibility that examination of the pairings of keywords and foils, in conjunction with contextual information, could allow target words to be identified without relying on the auditory input. Here, the ratio of targets and foils varied according to the number of potential targets for the phrase. When there was a single target word, that target was presented along with its single foil. When there were two potential target words, one of the two was selected at random and displayed together with one of its foils and both foils for the other potential target. When there were more than two potential targets, two were displayed along with one foil for each selected target and both foils for the nondisplayed target words. Thus, the possible display options were one target with one foil, one target with three foils, two targets with four foils, or two targets with six foils. The participant selected from the display the words that she or he believed had been in the phrase. When a foil was selected, the whole phrase was immediately replayed, with this process continuing until the one or two target words had been selected. At this point, the phrase was displayed orthographically and played out once again.

### Test Battery

The primary performance measure was sentence recognition in babble. Additional measures were included in an attempt to assess whether any posttraining improvement could be attributed to particular underlying aspects of speech perception. These measures included vowel and consonant identification in noise and memory span for digits in noise. Since memory for digits is affected by noise even when the digits are fully intelligible (Dallett, 1964), posttraining improvements in memory span may reflect benefits to cognitive aspects of speech perception. Both forward and backward digit span were measured (Wechsler, 1997). Forward digit span is regarded as a measure of verbal working memory, while backward digit span additionally requires the involvement of central executive processes (Pisoni, Kronenberger, Roman, & Geers, 2011).

#### Sentence recognition

Speech Reception Thresholds (SRTs) were measured in 20-talker babble for two types of sentences of differing complexity. BKB sentences contained three key words and Institute of Electrical and Electronical Engineers (IEEE) sentences (IEEE, 1969) contained five. As was the case in all the speech tests carried out, on each trial, the masker commenced 700 ms before the onset of the target speech and continued for 200 ms after the target offset, with cosine onset and offset ramps of 100 ms applied to the mixture. Participants’ spoken responses were scored by an experimenter. In each measurement run, the first of 20 sentences was presented at a signal-to-noise ratio (SNR) of +10 dB. In most cases, an adaptive procedure tracking 50% words correct was used in which SNR decreased if more than half of the key words were correctly identified and increased otherwise. Two participants scored at or below 80% correct on IEEE sentences in quiet, and in these cases, a performance level of 40% was tracked with SNR increased for 0 or 1 words correct, kept the same for 2 words correct, and decreased for 3 or more words correct. Regardless of the level tracked, a 10-dB change in SNR was used until the first reversal, 6.5 dB until the second reversal, and 3 dB for all subsequent reversals. SRTs were calculated as the mean SNR for the final even number of reversals with the 3 dB step size.

#### Vowel and consonant identification

Testing was conducted in speech-shaped noise at a fixed SNR, set for each individual participant. Speech-shaped noise was used because the information identifying individual phonemes occurs over a very short time frame and it was reasoned that fluctuations present in babble maskers might lead to undue variability in performance. Nine monophthongs in /bVd/ context were used: /æ/ (bad), /ɑː/ (bard), /iː/ (bead), /ɛ/ (bed), /I/ (bid), /ɜː/ (bird), /ɒ/ (bod), /uː/ (booed), and /ʌ/ (bud). Three tokens of each were presented in speech-shaped noise at a fixed SNR, so that each vowel test block contained 27 trials. 16 consonants [m n w l j b p d t g kʃs f z v] in /VCV/ format were tested. Each was presented in three different vowel contexts (/i/, /u/, and /ɑ/), resulting in a total of 48 trials per consonant test block. In both types of test, participants responded using a mouse to click on one of a set of orthographically labelled buttons displayed on a computer screen. In vowel tests, the buttons were labeled with the full /bVd/ words; in consonant tests, only the consonant was displayed (ʃ was displayed as “sh” and j as “y”).

#### Memory span for digits

Series of digits of increasing length were presented at a rate of one digit per second in speech-shaped noise at a fixed, individually determined SNR. At the end of a series, participants were required to type the sequence of digits into a box displayed on a computer screen. In different conditions, they were required either to enter the digits in the order that they were presented or in the reverse order. Testing began with a series containing two digits. Series length was increased every two trials until both trials of the same length were incorrectly responded to. Digits within a series were selected at random, without replacement, from the set 1 to 9.

#### Talkers

Sentence, vowel, and consonant tests were carried out using materials from one male and one female talker of standard Southern British English. To ensure that any benefits of training were generalizable and not talker specific, these talkers were different from those used in the training materials. The female talker was the same for all tests. Three different male talkers were used, one for BKB sentences, one for IEEE sentences, and one for both /bVd/ and /vCv/ materials. A single different male talker of standard Southern British English recorded the digits used in the memory span task.

### Equipment and Procedure

Both testing and training were implemented using custom MATLAB software (version 8.2.0.721, The Mathworks, R2013b). Testing was conducted in a sound-proof room at University College London using a Dell Latitude E6430 computer connected to a Fostex 6301B loudspeaker placed approximately 1 m in front of the participant. Stimuli were presented at a comfortable listening level (approximately 70 dB SPL). Participants were asked to set their CI speech processors and, where applicable, hearing aids, to their typical strategy for everyday listening situations and to ensure that the same settings were used for all test sessions. Two participants (S6 and S8) had routine appointments at their clinical CI center between pretraining test sessions, during which some adjustments to their processor maps were made.

To control for procedural learning, the test battery was typically performed four times prior to training—once in quiet and then three times in noise. For sentence tests, the order in which different sentence lists were used, over both pre- and posttraining tests, was counterbalanced across participants as far as possible. The different elements of the test battery were completed in a largely consistent order across participants and repetitions. The first SRTs obtained with BKB sentences were used as a basis for setting the individual SNRs used in digit span and vowel and consonant tests. The intention was to achieve initial vowel and consonant identification performance at a level where there was room for improvement without the task being dispiritingly difficult. For some participants, the SNR for vowel and consonant tests was adjusted following the first run in noise.

In the majority of cases, pretraining testing was spread over three test sessions at approximately weekly intervals. Due to time constraints, three participants were tested in two sessions, resulting in a slightly reduced number of tests. S2 and S8 completed vowel and consonant identification in noise on only two occasions and S2 and S9 were tested on digit memory span in noise only twice. Posttraining tests in noise were conducted the day after the final training session. All participants, other than S8, were also tested in noise approximately four weeks later, having not carried out training in the intervening period.

Training was performed in the participants’ own homes using a tablet PC (Dell Venue 11i Pro) which was loaned to the participant on completion of their final pretraining test session. The training software was run from a Dropbox folder, allowing progress to be monitored continuously. Sounds were presented either via direct connection between the tablet and the participant’s speech processor (S1 and S3) or via Sennheiser PX-100 supra-aural headphones, which are small and lightweight and could comfortably be worn at the same time as a speech processor and hearing aid. Presentation levels were set to a comfortable listening level prior to the participant taking away the tablet PC and were automatically set to the same level at the beginning of each training session. Participants were, however, able to adjust presentation level during a session.

Participants were requested to train for 30 min per day, 6 days a week for 4 weeks, making a total of 12 hr training. They were requested to carry out the training in a quiet room at a time when they were unlikely to be interrupted. Clicking on an icon on the tablet desktop launched a 30-min training session with a particular text. The session was divided into four blocks of 7.5 min which alternated between the male and female talker. The participant clicked on a button on the tablet screen to launch each block. Timings were controlled automatically by the tablet, though the participant could, of course, quit an ongoing session at any point. Participants were free to switch between the three texts as they wished over the course of training but, typically, texts were worked through in their entirety.

SNR was varied adaptively during training based on performance over sets of 10 consecutive phrases, with the aim of keeping the task challenging but not too difficult, which is widely believed to promote learning (e.g., Boothroyd, 2010). Separate adaptive tracks were maintained for each combination of talker and text, with an initial SNR of +10 dB. Performance was assessed in terms of the proportion of possible errors made (i.e., the total number of foils selected divided by the total number of foils displayed). If this proportion was greater than 0.15, then SNR was increased by 3 dB for the subsequent 10 phrases, otherwise it was decreased by 3 dB, with the exception that a minimum SNR (typically −2 dB) was imposed in order to guard against the possibility that contextual cues might allow very low error rates during some parts of a text. When SNR changed, both signal and noise levels were adjusted, keeping the overall level constant. At the start of each subsequent 7.5-min block with a given text and talker, the SNR was set to 3 dB above the last SNR used in the previous block with that combination.

## Results

### Pretraining Performance

High levels of speech perception performance in quiet were observed. Averaged across talkers, mean percentage words correct was 95.0% for BKB sentences and 84.0% for IEEE sentences. Mean vowel and consonant identification in quiet were 79.2% and 77.9%, respectively. There was little evidence of procedural learning over the course of pretraining speech testing in noise. Averaged over the different sentence materials, mean SRTs were 6.1 dB, 6.2 dB, and 6.1 dB for the first, second, and third test sessions, respectively. For the final two pretraining sessions, for which SNR did not vary within participants, mean vowel identification was 71% and 72%, while mean consonant identification was 57% and 55%. Mean forward digit span in noise was 5.6 and 6.1 for the final two sessions and mean backward digit span was 4.3 and 4.8. These means suggest that a degree of procedural learning for the digit span tasks may still have been occurring at the end of the pretraining period, although paired *t* tests did not show significant differences across the final two sessions for either forward, *t*(8) = 1.64, *p* = .139, or backward span, *t*(8) = 1.51, *p* = .169.

### Training

For most participants, there were only minor deviations from the intended timetable (Table 2). The two exceptions were S2 and S8. Due to unforeseen personal circumstances, S2 stopped carrying out training approximately a week into the intended 4-week training period, but was able to restart approximately four weeks later. Due to scheduling difficulties, S8 carried out training for nearly 5 weeks, rather than 4, and was unavailable for follow-up testing. Table 2 also shows the amount of training completed by each participant, both in terms of the number of 7.5 min training blocks started and the total number of phrases heard during training. Had participants followed exactly the suggested training regime, they would have completed 96 blocks. Only one participant, S4, was substantially below this number, while several were above it. The number of phrases heard during training varied from 2,216 to 3,161 with a mean of 2,692.

**Table 2.** Time Periods Between Test Sessions and Amount of Training Completed for Each Participant.

ID	Days between pre and posttraining tests	Days between posttraining and follow-up	Total training blocks started	Total phrases in training
S1	31	26	86	2,747
S2	56	36	94	2,216
S3	29	27	94	2,579
S4	28	28	76	2,184
S5	29	28	85	2,638
S6	28	30	106	2,870
S7	28	28	101	2,802
S8	34	NA	114	3,161
S9	29	32	110	3,030

**Figure 1.**
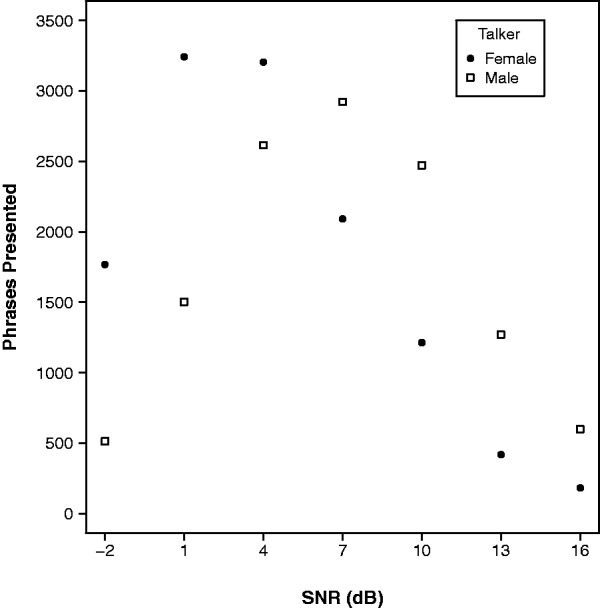
Number of phrases presented at each SNR during training, summed across participants.

Figure 1 shows the number of phrases presented at different SNRs during training with each talker, summed across all participants. The spread of phrases across SNRs suggests that the adaptive procedure operated satisfactorily, although the proportion of phrases at lower SNRs is considerably greater for the female talker. This indicates that the female training talker was more intelligible than the male and suggests that a slightly lower minimum SNR for the female talker might have been appropriate.

**Figure 2.**
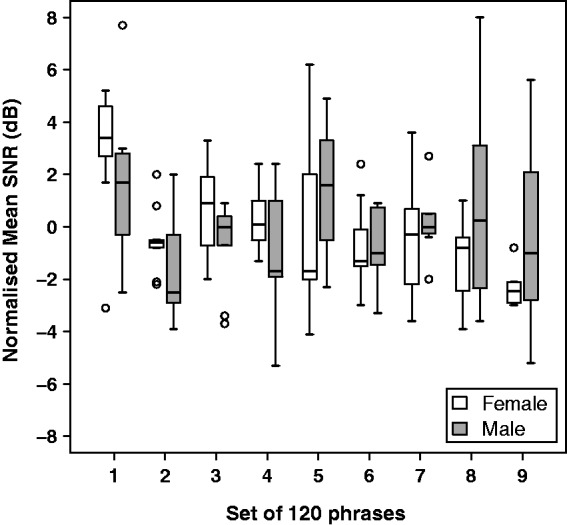
Boxplot of normalized mean SNR over successive completed sets of 120 sentences for each training talker. To indicate progress over the course of training, mean SNRs obtained for each participant were normalized by subtracting the mean SNR over all included sets for that participant and talker.

The difficulty of identifying the key words likely varied quite substantially across different phrases and different sections of a text, making it difficult to get an accurate picture of how performance changed over time. However, in order to get some indication of progress over the course of training for each participant, mean SNRs were calculated over successive sets of 120 phrases with a given talker. Since SNR was always set to +10 dB at the beginning of a text, the initial set of phrases after a change of text were omitted, as were incomplete sets at the end of a text. To highlight variation over the course of training, rather than across-participant variability, the mean SNRs obtained for each participant were normalized by subtracting the mean SNR over all included sets for that participant and talker. As shown in Figure 2, for both talkers, there was a substantial decline in mean SNR between the first and second sets of 120 phrases, but little consistent change subsequently.

### Effects of Training

#### Sentence recognition

Table 3 shows SRTs averaged across participants before and after training for each of the four sentence tests. For each type of sentence material, performance improved relative to the final pretraining session, both when assessed immediately after training and at the 4-week follow-up. As might be expected given their greater complexity, SRTs were generally higher for IEEE than for BKB sentences. For the female sentences, where the talker was the same for both sentence types, SRTs were between 3 and 5 dB lower for BKB sentences across different test sessions. Male talkers differed across sentence types, but for both BKBs and IEEEs, performance was better with female than male speech.

**Table 3.** Mean SRTs in dB Before and After Training, for Each Sentence Test.

Test	Pretraining	Posttraining	Follow-up
BKB Female	2.9 (2.6)	2.2 (2.0)	1.5 (1.8)
BKB Male	5.5 (1.7)	4.1 (1.4)	3.4 (1.7)
IEEE Female	8.0 (3.3)	5.4 (2.6)	4.6 (1.6)
IEEE Male	8.2 (4.5)	6.3 (3.3)	6.9 (3.4)
Mean	6.1 (2.1)	4.5 (1.8)	4.1 (1.6)

*Note.* Lower SRTs mean better performance. Standard deviations are shown in parentheses. Note that pre- and posttraining means are derived from nine participants, but S8 is missing from follow-up.

**Figure 3.**
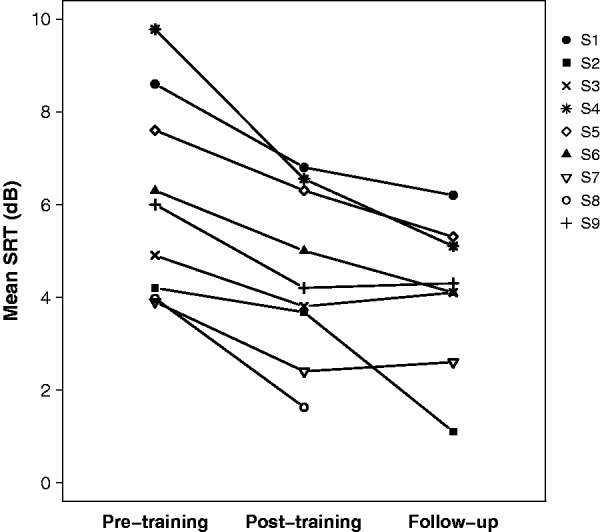
SRTs averaged across sentence material before and after training for each participant.

Data were analyzed with a linear mixed-effects model with participants as a random factor and with fixed factors of test time (pretraining, posttraining, and follow-up) and sentence condition (BKB female, BKB male, IEEE female, and IEEE male). The use of a mixed-effects model allowed the incomplete data set from S8 to be included. There were significant main effects of both test time (*F* = 11.01, *p* < .001) and sentence condition (*F* = 27.73, *p* < .001), but no significant interaction (*F* < 1). Figure 3 shows individual participants’ SRTs before and after training, averaged across sentence condition. For all participants, posttraining mean SRT was lower than pretraining SRT, with the improvement ranging between 0.5 and 3.2 dB. Mean SRTs at follow-up either remained very similar to posttraining or continued to improve. Differences between pretraining and follow-up ranged between 0.8 and 4.7 dB. Bonferroni-corrected pairwise comparisons showed that posttraining and follow-up SRTs were both significantly different from pretraining SRTs, but did not differ significantly from each other.

#### Vowel and consonant identification

**Figure 4.**
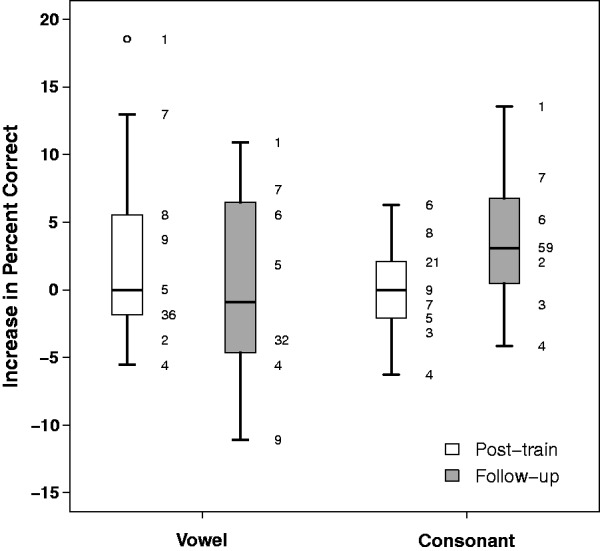
Boxplot of changes in vowel and consonant identification averaged across talker relative to pretraining performance. The numbers to the side of each box show individual data.

There were only small changes in mean vowel and consonant identification after training (Table 4 and Figure 4). Mean improvement in vowel identification was 3.1 percentage points immediately after training and 0.2 percentage points at follow-up. For consonant identification, mean improvement was 0.2 percentage points at the posttraining test and 3.8 percentage points at follow-up. Logistic mixed effects analysis of vowel identification averaged across talker showed no significant effect of test time (*F* < 1). For consonant identification, there was a significant effect of test time (*F* = 3.49, *p* = .048). However, Bonferroni-corrected pairwise comparisons showed no significant differences between any particular pair of test times.

**Table 4.** Mean Percentage Correct Vowel and Consonant Identification Before and After Training.

		Pretraining	Posttraining	Follow-up
Vowel	Female	75.3 (17.3)	77.4 (11.3)	76.9 (7.1)
	Male	69.2 (12.1)	73.3 (11.7)	70.8 (12.9)
	Mean	72.2 (13.7)	75.3 (9.3)	73.9 (8.9)
Consonant	Female	54.9 (10.9)	58.6 (12.6)	57.3 (9.0)
	Male	54.7 (15.3)	51.4 (12.3)	59.1 (11.1)
	Mean	54.8 (12.5)	55.0 (12.3)	58.2 (9.2)

*Note.* Standard deviations are shown in parentheses. Note that pre- and posttraining means are derived from nine participants, but S8 is missing from follow-up.

Some individual participants did show quite sizeable shifts in performance in either direction. However, these differences did not appear to be related to changes in sentence recognition. For example, S4 had the largest improvement in sentence recognition, yet showed poorer performance after training for both vowel and consonant identification. Conversely, S1 and S7 showed the largest increases in phoneme identification, but did not show especially large improvements in mean SRT.

#### Digit span in noise

**Figure 5.**
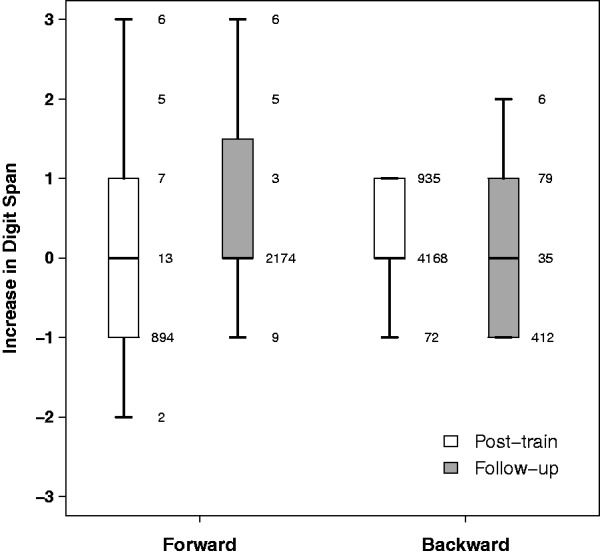
Boxplot of changes in forward and backward digit span in noise relative to pretraining performance. The numbers to the side of each box show individual data.

As shown in Table 5, there were small increases in mean digit span in noise after training for both forward and reverse order of recall. Mean improvement in forward digit span was 0.1 immediately after training and 0.6 at follow-up. For backward digit span, mean improvement was 0.1 at both test intervals. Linear mixed effects analysis showed no significant effect of test time in either case (*F* < 1 in both cases). As with phoneme identification, there was considerable individual variability (Figure 5), particularly for forward digit span, but no indication that this variability was related to changes in sentence recognition.

**Table 5.** Mean Digit Span in Noise Before and After Training.

	Pretraining	Posttraining	Follow-up
Forward	6.1 (1.4)	6.2 (0.8)	6.6 (1.2)
Reverse	4.8 (1.1)	4.9 (1.1)	4.9 (1.0)

*Note.* Standard deviations are shown in parentheses. Note that pre- and posttraining means are derived from nine participants, but S8 is missing from follow-up.

## Discussion

After 12 hr of computer-based connected-text speech in noise training, sentence recognition in noise was significantly improved. Averaged over participants and sentence materials, SRTs at the follow-up test session were around 2 dB better than at the final pretraining session. However, other than a minor effect on consonant identification, there was no clear evidence of significant improvement after training in phoneme identification and no significant improvement in either forward or backward digit span. Compliance with the requested training regime was good and while participants’ attitudes were not formally assessed, they generally reported that they had enjoyed the training, supporting the idea that the use of a connected text helps to keep the task engaging. While these data are encouraging, some important caveats must be considered.

Since sentence recognition did not improve over the repeated pretraining test sessions, it appears unlikely that this improvement derives from procedural learning. However, the absence of clear posttraining improvement in the secondary outcome measures gives no indication of which factors did underlie the observed improvements in sentence recognition. Identifying the underlying sources of improvement would be helpful both in relation to optimizing the training method and in considering the extent to which the observed improvements are likely to generalize to real-world situations. The small number of participants in the current study clearly limited the extent to which relationships between different outcome measures could be determined. The contribution of different underlying abilities may vary across individuals, so that, for example, for some individuals, improved sentence recognition after training may be associated with better phoneme identification with little change in cognitive abilities, while for others, cognitive skills are improved with no change in phoneme perception. This could result in significant changes at the group level being observed for sentence recognition but not for any particular underlying ability, as was the case here.

Speech recognition in adverse conditions draws on a range of cognitive abilities (e.g., Peelle, 2018) and it is clearly also possible that posttraining improvements in sentence recognition were mediated by cognitive skills that were not captured by the digit span in noise tests used here. One possibility is suggested by recent work investigating the role of semantic context in reducing listening effort. Winn (2016) compared the pupillary response for predictable versus unpredictable sentences for CI users, normal hearing listeners, and normal hearing listeners presented with CI simulations. Of particular interest here is the inference that cognitive processing related to the use of contextual cues could occur well after the end of the stimulus presentation. It was suggested that this might represent a form of “perceptual restoration.” For example, a listener might mistakenly perceive the word “bread” as “bird” but correctly report it as “bread” after subsequently hearing “made from wheat” at the end of the sentence. Given the nature of the present training, in which the ongoing narrative typically provides substantial contextual cues and the task involves selecting from among similar sounding alternatives, it seems plausible that improvements in this kind of perceptual restoration process may contribute to the observed improvements in sentence recognition. While this account can only be speculative, if correct, it has implications for the generalizability of the training benefit, since, as Winn (2016) notes, in many real-world communicative situations, the gaps between successive utterances may be too short for perceptual restoration to occur.

A further consideration is that while the target talkers used in all outcome measures differed from those used in training, so that benefits of training could not derive from increased familiarity with a particular talker, sentence recognition was tested with the same 20-talker babble used in training, whereas other testing was conducted in speech-shaped noise. It is therefore possible that the fact that significant improvement after training occurred for sentence recognition but not for the secondary measures arose in part from participants learning in some way to lessen the impact of the babble. There is some previous evidence of masker-specific effects in speech in noise training. Van Engen (2012) found that sentence recognition in babble for normally hearing listeners was better after short-term training in the same babble than after training in speech-shaped noise. However, note that in addition to other substantial differences from the current study, the babble in that case contained only two talkers, so that individual voices would have been distinguishable and informational masking would have played a major role. While the extent to which the present training benefits were due to consistency of the masker between training and testing cannot be determined, it would appear preferable in general to use a range of different realistic maskers during training.

Perhaps also of interest is whether posttraining improvement in sentence recognition may have been influenced by overlap between key words in sentences and target words in training. Examination of the occurrence of sentence key words in the training texts suggested that the proportion of key words that occurred at least once during training was around 0.6 for BKB sentences and 0.5 for IEEE sentences. Note that this calculation is based on all potential target words in training phrases, not just those that the participant was required to identify. For key words that appeared at least three times during training, the proportions were approximately 0.4 for BKBs and 0.3 for IEEEs. However, the extent to which this substantial overlap between test and target words contributed to the benefit observed cannot be determined. In any case, given the nature of the method, a considerable degree of overlap seems inevitable.

A further issue to consider is the possible role of broader psychological factors. Participants invested considerable time and effort in carrying out the training and posttraining performance may have been enhanced due to increased motivation and expectation of success. The fact that improvement was found only in sentence recognition and not in other posttraining tests may count against such an explanation, but it cannot be ruled out. Assuming that such an effect extended beyond the test environment and reflected a generally more confident and motivated approach to listening to speech in adverse conditions, it could represent a useful indirect benefit of training even in the absence of improvement in other aspects of speech perception.

Despite these caveats, these data support the idea that computer-based connected-text training has potential to be a clinically useful intervention. All participants had at least 10 months experience with their CIs before taking part and some had considerably more. Within this small data set, there was no evidence of an effect of duration of CI experience on the amount of benefit derived from training. For the four participants with up to 12 months experience, the mean improvement in SRT was 1.7 dB posttraining and 2.5 dB at follow-up, while for the three participants with 24 months or more experience, the corresponding values were 1.7 dB and 1.8 dB. It would, however, be of interest to see whether larger benefits would occur if training were implemented sooner after implantation.

## Conclusions

Moderate amounts of computer-based connected-text training carried out at home resulted in significantly improved sentence recognition in babble in a group of experienced CI users. No significant improvements were found for vowel or consonant identification or for a digit span in noise task that was intended to reflect some of the important cognitive abilities involved in speech perception in adverse conditions. Further research would be required to identify the underlying sources of improvement and to assess the extent to which benefits of training generalize to real-world situations. However, the present data suggest that the connected-text training approach has potential as a clinical intervention.
